# Depth shapes microbiome assembly and network stability in the Mariana Trench

**DOI:** 10.1128/spectrum.02110-23

**Published:** 2023-12-12

**Authors:** Yi Li, Jinjun Kan, Feilong Liu, Kaiyue Lian, Yantao Liang, Hongbing Shao, Andrew McMinn, Hualong Wang, Min Wang

**Affiliations:** 1 College of Marine Life Sciences, Institute of Evolution and Marine Biodiversity, Frontiers Science Center for Deep Ocean Multispheres and Earth System, and Key Lab of Polar Oceanography and Global Ocean Change, Ocean University of China, Qingdao, China; 2 UMT-OUC Joint Center for Marine Studies, Qingdao, China; 3 Microbiology Division, Stroud Water Research Center, Avondale, Pennsylvania, USA; 4 Institute for Marine and Antarctic Studies, University of Tasmania, Hobart, Tasmania, Australia; University of Mississippi, University, Mississippi, USA

**Keywords:** microbiomes, community complexity and stability, Mariana Trench, upper bathypelagic waters, hadal waters

## Abstract

The Mariana Trench, a stable marine ecosystem minimally impacted by human activities, has the greatest vertical depth in the ocean. Depth gradients profoundly influence the physical, chemical, and biotic conditions in the deep oceans. Therefore, elucidating the depth-related effects on microbiome assembly and interaction is integral to understanding marine hadal ecosystems. In this study, eight samples were collected along vertical gradients to compare the assembly and associations of bacteria, archaea, and microeukaryotes, in the Marina Trench using high-throughput sequencing. There is no significant difference in the niche breadth occupied by bacteria, archaea, and microeukaryotes, indicating that microorganisms from the surface to the hadal water of the trench are well adapted to this environment. Stochastic processes significantly influenced the distribution of bacteria and microeukaryotes as well as archaea when the phylogenetic distance was considered. Although diversities of all three microbial domains were consistent across depth, distinct bacterial and archaeal community structures occurred between hadal waters (3,699–8,727 m) and upper bathypelagic waters (≤1,000 m). Bacteria and archaea in upper bathypelagic zones were more affected by a random process and exhibited higher phylogenetic distance than those in hadal waters. In contrast, stochasticity in assembly and phylogenetic distance of microeukaryotes remained unchanged. Bacteria made vital contributions to the stability of trench microbiomes, while consistent community stability was observed between the upper bathypelagic and hadal zones. Our results reveal the complexity of community assembly and associations of multi-domain microbiomes in hadal ecosystems and also highlight the differential adaptations and contributions of prokaryotes vs. microeukaryotes to microbiome community stability and robustness.

**IMPORTANCE:**

Exploring microbial interactions and their stability/resilience from the surface to the hadal ocean is critical for further understanding of the microbiome structure and ecosystem function in the Mariana Trench. Vertical gradients did not destabilize microbial communities after long-term evolution and adaption. The uniform niche breadth, diversity, community complexity, and stability of microbiomes in both upper bathypelagic and hadal waters suggest the consistent roles of microbiomes in elemental cycling and adaptive strategies to overcome extreme environmental conditions. Compared with microeukaryotes, bacteria and archaea play a pivotal role in shaping the stability of the hadal microbiome. The consistent co-occurrence stability of microbiomes across vertical gradients was observed in the Mariana Trench. These results illuminate a key principle of microbiomes inhabiting the deepest trench: although distinct microbial communities occupy specific habitats, the interactions within microbial communities remain consistently stable from the upper bathypelagic to the hadal waters.

## INTRODUCTION

The Mariana Trench represents the deepest known site in the Earth’s oceans (1). Its hadal zone is characterized by multiple extreme environmental conditions (2). Nevertheless, abundant microorganisms and metabolic activities have been discovered in the hadal water (3 – 5). Hadal microbial communities play critical roles in organic matter remineralization, carbon storage and burials, and food web dynamics (6). Systematically investigating the composition, distribution, and interactions of hadal microbiomes will advance our understanding of these unique deep ocean ecosystems and their ecological functions.

Marine microbiomes (consisting primarily of bacteria, archaea, microeukaryotes, and virus) are the dominant drivers of biogeochemical processes in the ocean (7 – 10). These highly diverse microbial communities play vital roles in oceanic food webs and global biogeochemical cycles (11, 12). From the epipelagic zone down to the hadal depths, microbial diversity and community structure have been extensively characterized in the Mariana Trench. T. Nunoura et al. (4) revealed that the hadal waters (HW) harbor previously unknown trench-specific bacterial communities, distinct from the abyssal microbial communities. Z. Xu et al. (13) and J. Tian et al. (14) described the abundance and distribution patterns of microbial communities in the Mariana Trench. Their results demonstrate depth-dependent variations in microbial genetic signatures and community structures from the surface to the hadal zone, revealing vertical transitions of trench microbiomes. This extends our understanding of hadal microbial community dynamics. Additionally, X. Y. Zhu et al. (15) examined the diversity and metabolic potential of microbial eukaryotes, discovering clear depth-dependent distributions and associated metabolic pathways. Bacterial populations in the bottom waters of Challenger Deep differed from those in Sirena Deep, indicating potential impacts from distinct chemical conditions between these two sites (16). Additionally, the deepest known chemolithoautotrophic microbial mats were discovered from filamentous structures covering both talus and outcrop in the ocean (17). These studies have provided important insights into the vertical distribution of microbial communities in the Mariana Trench. However, microbiome interactions among different domains and adaptation mechanisms to the abyssal environment remain poorly understood and undefined.

Bacteria, archaea, and microeukaryotes are ubiquitous in aquatic environments, and interactions between these microbial communities are crucial to hadal ecosystems. A promising approach to elucidate how environmental gradients affect community complexity, interactions, and stability is co-occurrence network analysis (18 – 20). Microbial co-occurrence networks enable prediction of hub species and potential species interactions (21). For example, co-occurrence patterns between bacteria and eukaryotes in the hyphosphere imply processes involving cross-kingdom biotic interactions and niche sharing between nonmycorrhizal bacteria and eukaryotes (22). Richter et al (2019)^23^ demonstrated greater biogeographic structuring of smaller planktonic organisms like bacteria compared with larger metazoans or protists. Currently, interactions between bacteria, archaea, and microeukaryotes in the Mariana Trench remain poorly characterized. How these interactions facilitate adaptation of each microbial group to the abyssal environment also remains unclear.

In recent years, with growing environmental pressures such as anthropogenic activities and climate change, ecosystem stability and microbial interactions have garnered extensive attention. Even in the deep ocean, far from land, environments are being affected by human activities. For example, antibiotic resistance genes (ARGs) from anthropogenic pollution can settle to the bottom of the Mariana Trench, where dispersion is constrained by the unique topographic features (i.e., the funnel-like shape) (24). Similarity, the Yap Trench accumulates marine snow, marine excrement, and anthropogenic pollutants (i.e., antibiotics pollutants) driven by settling mechanisms at extreme depths (25). H. Su et al. (26) tested the probability of anthropogenic pollution leading to the occurrence of ARG in seawater and sediments of the Yap Trench, clarifying its potential anthropogenic influence. Additionally, climate warming significantly enhances the complexity and stability of molecular ecological networks in grassland soil microbial communities (27). In contrast, naturally occurring soil microbiomes in a Florida scrub ecosystem exhibit network properties of unstable communities under persistent stress (28). We also found that microbial networks in the Chesapeake Bay demonstrate spatiotemporal variations in response to natural and anthropogenic gradients (29). Numerous network properties and statistical matrices can successfully predict microbiome network complexity (e.g., network size, connectivity, average clustering coefficient, and relative modularity) and stability (e.g., fragmentation, robustness, and vulnerability) of microbiomes (27, 28, 30). For example, modules in co-occurrence networks may indicate ecological processes shaping community structure like niche filtering and habitat preference (20). Elucidating the complexity and stability of microbiome networks will provide insights into community-based microbial distributions and ecosystem functions, which remain largely unexplored in hadal ecosystems such as the Mariana Trench.

To investigate microbiome interactions in the Mariana Trench, we characterized microbial vertical profiles from the epipelagic to the hadal zones using high-throughput sequencing of 16S rRNA and 18S rRNA genes. This allowed detailed deciphering of microbiome structures for downstream analyses investigating the distribution and interactions between bacteria, archaea, and microeukaryotes in the Mariana Trench. Our aims are as follows: (1) explore the distribution and niche breadth of bacteria, archaea, and microeukaryotes in the hadal ecosystems; (2) compare phylogenetic characteristics and stochastic processes of microbiome responses to environmental gradients; (3) explore microbiome interactions between upper bathypelagic and hadal waters in the Mariana Trench; and (4) investigate vertical assembly and stability of ocean microbiomes under persistent, strong environmental filtering. These results will improve our understanding of the assembly and associations of deep-sea microbial communities, provide an integrated perspective on vertical responses of marine microbiomes to spatial scales, and further help predict microbial community responses to future environmental changes in the Mariana Trench.

## RESULTS

### Diversity and niche breadth of microbiomes

**Fig 1:**
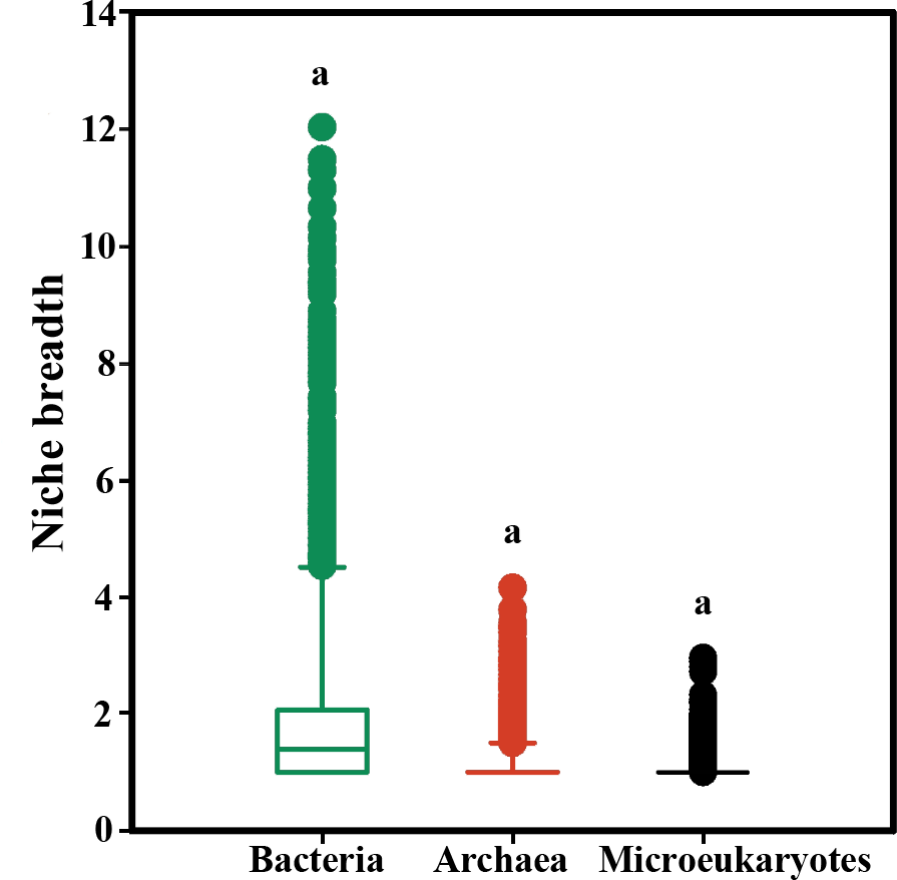
The environmental niche breadth for bacteria, archaea, and microeukaryotes in the trench. Values with superscript letters a and b are significantly different across columns (*P* < 0.05).

Diversity (richness, Shannon-Wiener, Simpson, and Pielou’s evenness) of total bacterial and archaeal communities or abundant and rare subgroups did not differ over depth or between attached and free-living microbiomes (Table 1). Similar results were also found in microeukaryotes, except richness and chao1 of abundant subgroups and Simpson of rare groups significantly correlated with the depth gradients (Table 1). Microbial community diversity was primarily contributed by and correlated with the rare subgroups. By calculating the niche breadth for the three domains, we found no significant differences in niche breadth occupied by bacteria, archaea, and microeukaryotes from the surface to hadal waters in the Mariana Trench (Fig. 1). The uniform distribution consistent diversity and niche breadth among microbiomes suggest replenishment by taxa well adapted to vertical gradients in trench.

**TABLE 1:** Two-way ANOVA showing the effects of depth and sampling pore size on the diversity of different microbial groups^*a*^

		Bacteria			Archaea			Microeukaryotes				
		Total	Abundant	Rare	Total	Abundant	Rare	Total	Abundant		Rare	
Depth	Richness	2.05	0.86	2.71	0.04	0.12	0.05	2.72	10.6	*	3.38	
	Shannon-Wiener	2.42	0.04	1.77	0.13	0.01	0.79	0.98	3.77		5.33	
	Simpson	0.8	0.14	1.02	0.69	0.16	1.96	0.82	1.79		7.63	*
	Pielou’s evenness	2.15	0	2.56	2.73	3.45	0.03	0.4	0.26		1.69	
Pore size	Richness	0.77	0.14	0.74	0.16	0.23	0.65	1.48	0.18		1.36	
	Shannon-Wiener	2.53	3.5	1.38	0.14	1.87	0.13	0.59	1.83		1.48	
	Simpson	1.77	2.05	1.72	1.23	3.64	0.01	0.09	0.47		1.41	
	Pielou’s evenness	2.74	4.07	0.09	1.38	5.1	0.26	0.22	0.44		0.95	
Depth × pore size	Richness	0.38	0	0.43	0.11	1.16	0.83	0.05	2.84		0.03	
	Shannon-Wiener	0.3	0.01	0.31	0.22	0.01	0.36	0.09	4.13		0.29	
	Simpson	0.16	0.01	0.16	1.58	1.05	0.07	1.01	3.2		0.54	
	Pielou’s evenness	0.2	0	0.51	0.55	3.75	1.41	0.41	3.42		0.04	

^*a*^ ***, *P* < 0.001; **, *P* < 0.01; *, *P* < 0.05.

### Distribution and phylogenetic characteristics of microbiomes

**Fig 2:**
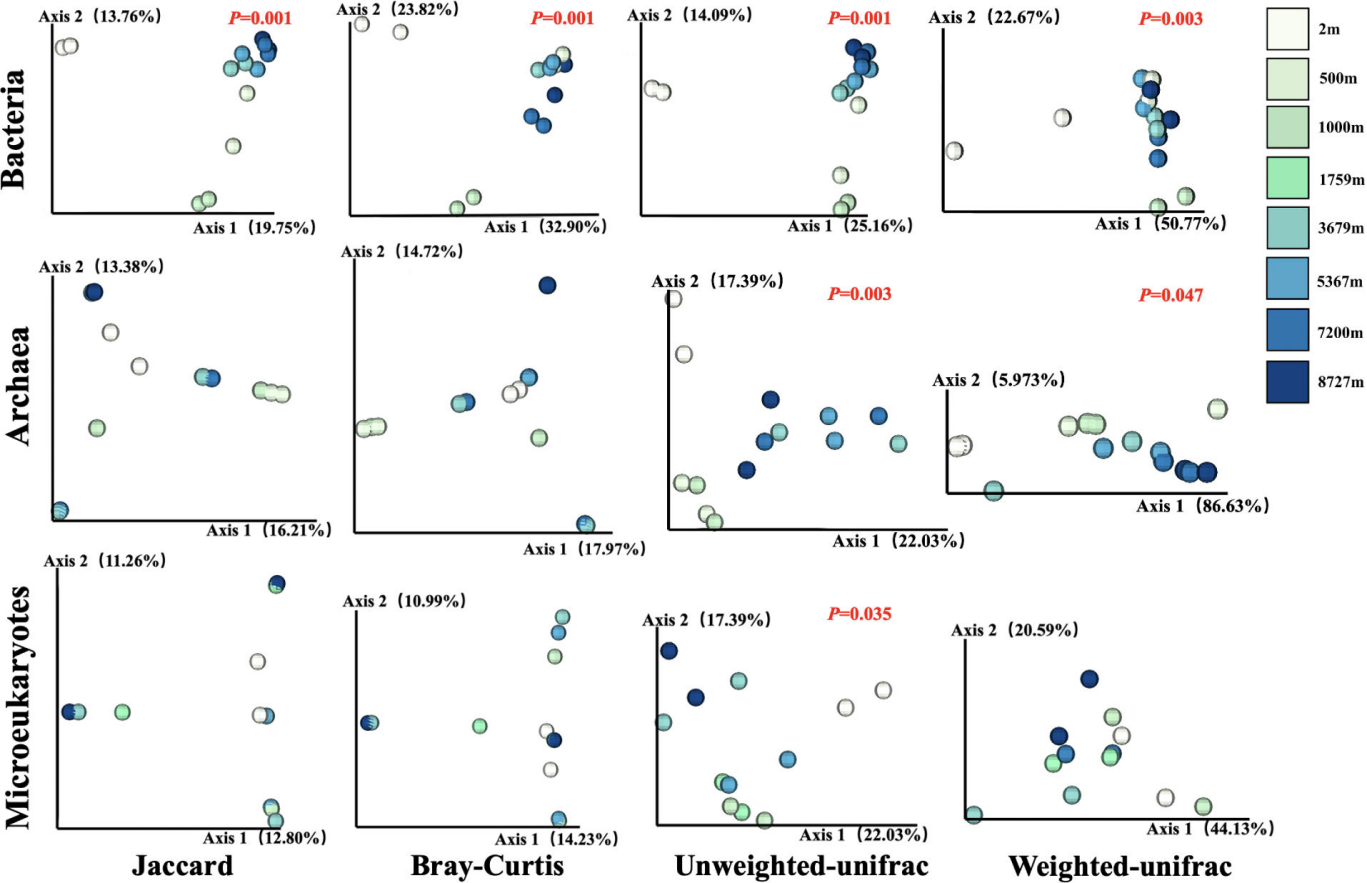
Nonmetric dimensional scaling (NMDS) distribution patterns of microbiomes in the Mariana Trench: top, bacteria; middle, archaea; and bottom, microeukaryotes. The distance/dissimilarities are calculated based on Jaccard, Bray-Curtis, Unweighted-UniFrac, and Weighted-UniFrac.

Vertical gradients significantly affected microbiome community composition from the surface to hadal waters in the Mariana Trench (Fig. 2). Bacterial communities exhibited consistent response to depth gradients based on four types of β-diversity metrics. However, clear vertical patterns of archaeal communities and microeukaryotes emerged only when considering phylogenetic distances between taxa, or both phylogenetic distances and occurrences at each site were included, respectively. In addition, analysis of similarities (ANOSIM) tests revealed vertical separation of microbiomes in the trench, with significant differences identified between upper bathypelagic and hadal zones (*P* < 0.01).

**Fig 3:**
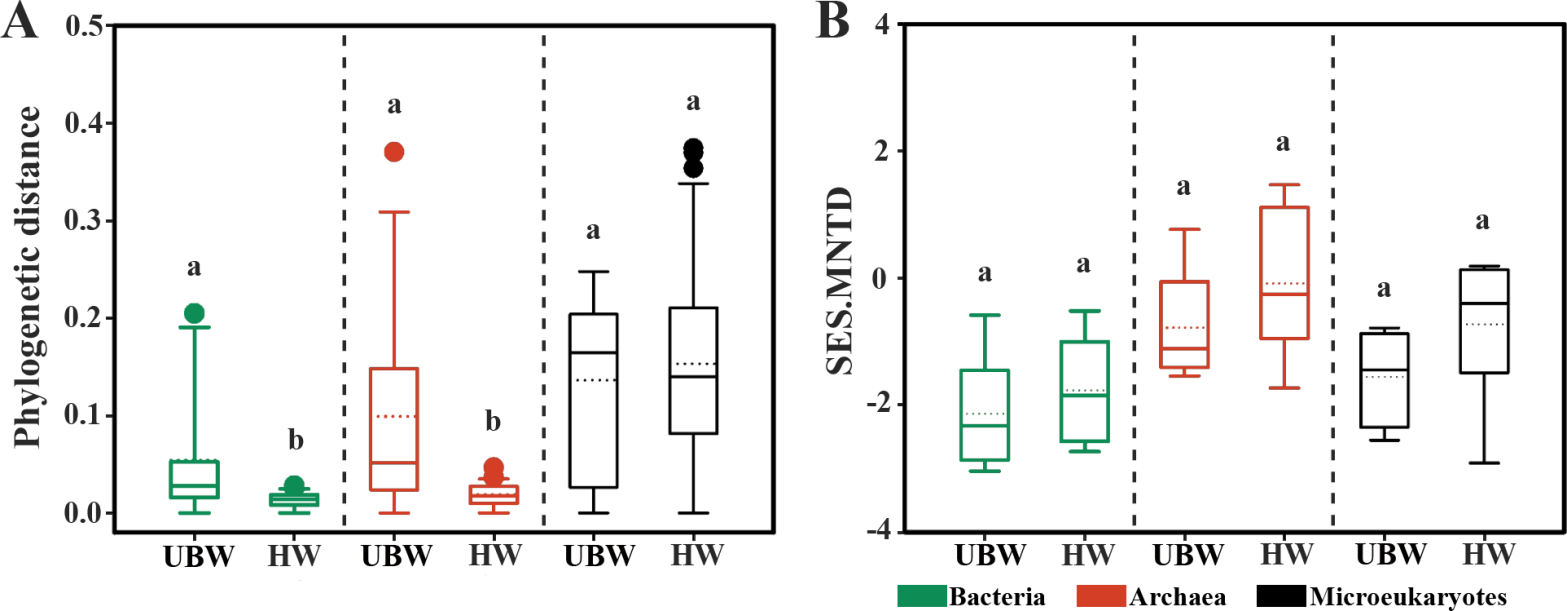
Phylogenetic distances (**A**) and SES.MNTD values (**B**) of bacteria, archaea, and microeukaryotes from UBW to HW in the trench. Values with superscript letters a and b are significantly different across columns (*P* < 0.05).

The phylogenetic distances of bacteria and archaea in upper bathypelagic waters (UBW) were significantly higher than those in the hadal ocean, where the phylogenetic distances for microeukaryotes were similar across depths (Fig. 3A). These results imply greater phylogenetic diversity within prokaryotic communities in upper bathypelagic waters compared with the hadal waters. No significant differences in SES.MNTD were found between upper bathypelagic and hadal layers for bacteria, archaea, or microeukaryotes (*P* > 0.05) (Fig. 3B). The negative SES.MNTD values indicated phylogenetic clustering within communities that were more closely related than expected by chance. This suggests environmental filtering likely shapes microbiome assembly in the Mariana Trench.

### Responses of microbiomes to environments

**Fig 4:**
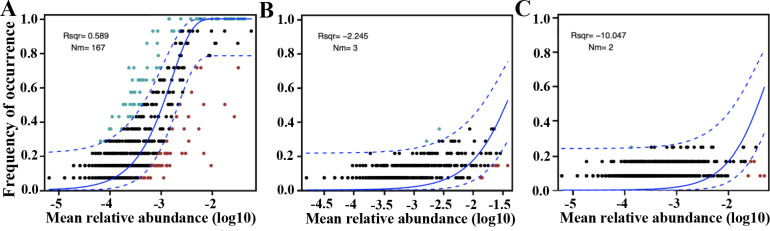
Neutral community model showing the potential importance of random processes for bacterial (**A**) and archaea (**B**) and microeukaryotic microbiomes (**C**) in the Mariana Trench. The predicated occurrence frequency is shown as a solid blue line, and dashed blue lines represent 95% confidence intervals around the model prediction. Red and cyan dots indicate the operational taxonomic unit that occur less and more frequently than given by the model. *m* indicates the metacommunity migration rate and *R* ^2^ value indicates the fit to this model.

The neutral community model was applied to determine the potential importance of random processes in microbiome assembly in the Mariana Trench (Fig. 4). The relations between bacterial taxa occurrence frequency and relative abundances were successfully estimated with high interpretation rates (*R* ^2^), suggesting the random processes contribute greatly to bacterial community assembly (Fig. 4A). In contrast, low interpretation rates occurred in archaea and microeukaryotes, indicating additional assembly mechanisms like the deterministic process also influence the community structure (Fig. 4B and C).

**Fig 5:**
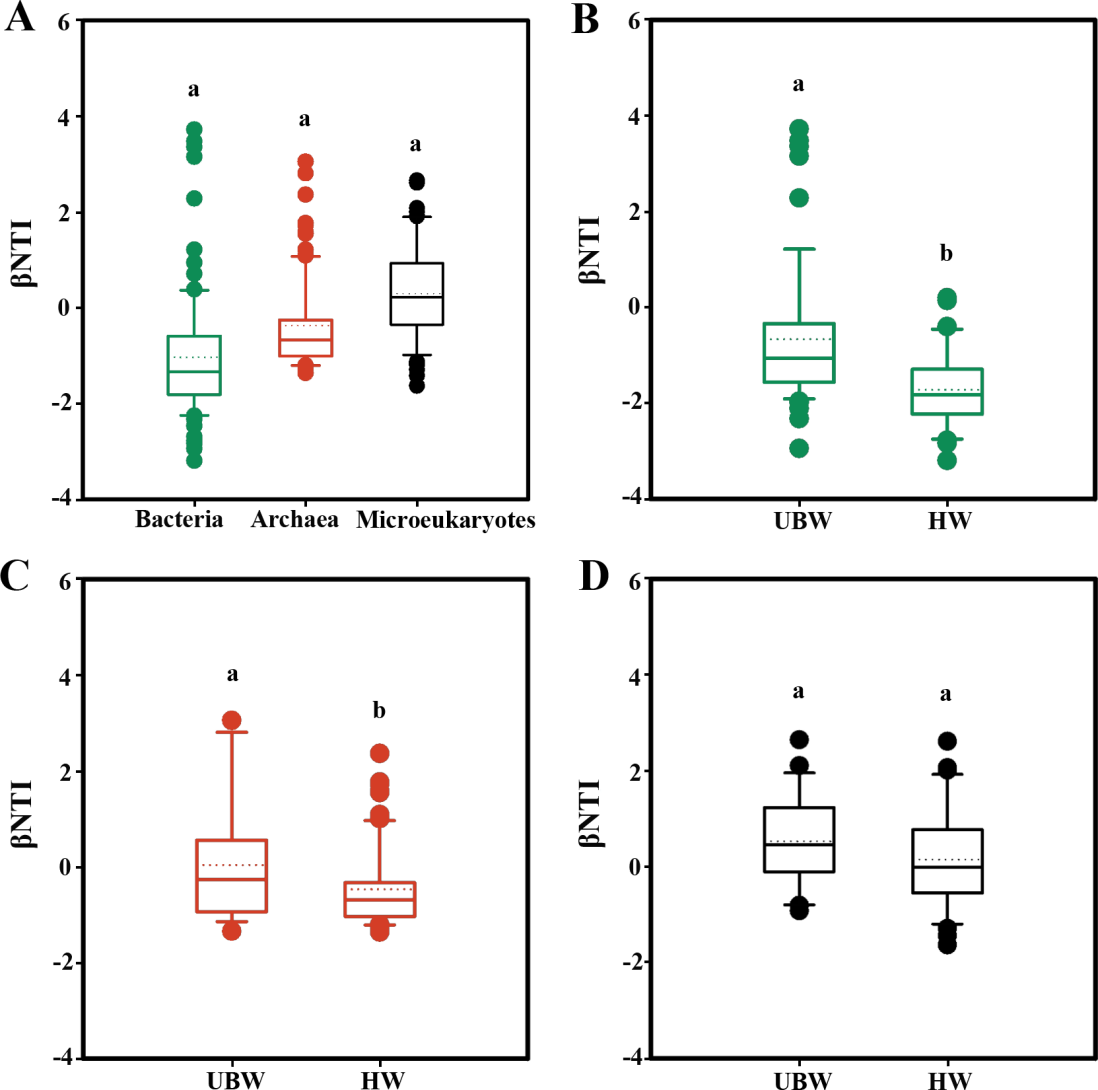
The differences in taxonomic and phylogenetic diversity of microbiomes in the Mariana Trench based on the null model-based Bray-Curtis-based βNTI statistical analysis results. βNTI values of bacteria, archaea, and microeukaryotes (**A**); βNTI values of bacteria (**B**); archaea (**C**); and microeukaryotes (**D**) in the UBW to HW, respectively. Values with superscript letters a and b are significantly different across columns (*P* < 0.05).

Interestingly, after incorporating phylogenetic distances in β-nearest taxon index (βNTI) analysis, random processes were found to influence prokaryotic and eukaryotic community distributions, as evidenced by βNTI values within −2 to 2 for all three domains (Fig. 5A). Similarly, microbial distributions were affected by random process in both upper bathypelagic and hadal waters (Fig. 5B through D). Moreover, βNTI values were significantly higher for bacterial and archaeal community assembly in upper bathypelagic and hadal waters. This indicates vertical gradients from surface to the hadal waters decreased stochasticity in bacterial and archaeal community assembly, while stochasticity in microeukaryotic assemblies remained unchanged between upper bathypelagic and hadal oceans.

Multivariate regression tree (MRT) analysis confirmed the impact of vertical gradients on microbiome distributions with depth in the trench. Depth (29.4%), dissolved oxygen (DO) (17.6%), and temperature (7.0%) were the major drivers to shape the distribution of bacterial communities (Fig. S2), while the accumulative variances of microeukaryotes were mainly explained by factors including depth (21.6%), phosphorus (12.4%), and temperature (6.8%) (Fig. S2). Archaeal community variances were mainly explained by pH (13.9%), depth (12.3%), temperature (7.5%), and DO (6.4%). MRT results clearly demonstrated that depth was the most important factor driving vertical variations across all three microbiome domains, along with other environmental gradients.

### Complexity of microbiome co-occurrence associations

**Fig 6:**
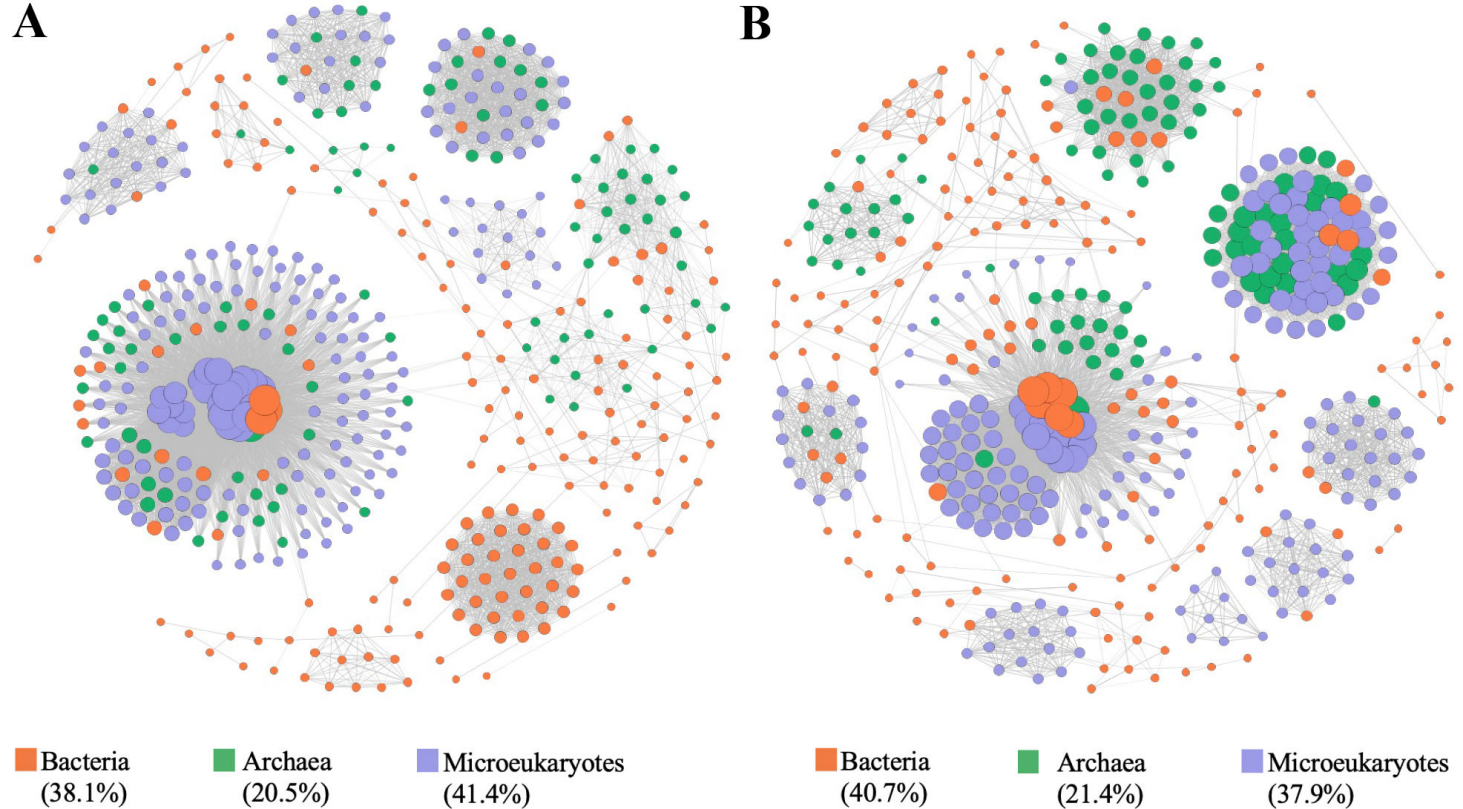
Co-occurrence networks of microbiomes in the upper bathypelagic (**A**) and hadal (**B**) oceans.

Microbiome (including bacteria, archaea, and microeukaryotes) co-occurrence networks in the trench were constructed based on the Pearson correlations of log-transformed amplicon sequencing variation (ASV) relative abundances, using a random matrix theory approach. The networks of microbiomes were comprised of highly connected microbial taxa with both positive and negative relations, and densely connected groups formed a clustered topology with comparable variations between upper bathypelagic and hadal waters (Fig. 6). Topological properties commonly used in network analysis were calculated (Table S2). The network of upper bathypelagic waters contained 483 nodes and 7,575 edges (average degree of 31.4), while the network of hadal waters had 467 nodes and 6, 808 edges (average degree of 29.2) (Table S2). More microbial taxa and associations occurred in upper bathypelagic waters compared with hadal waters. Modularity indexes exceeding 0.4 in the upper bathypelagic and hadal waters indicated modular structures, especially in hadal waters (Table S2) [76]. Modules are densely correlated microbial groups governed by trench ecological processes like conserved inter-species communications, serving as indicators of habitat partitioning/preferences [67].

The clustering coefficients which represent the degree to which nodes tend to cluster together and the numbers of clusters and components were almost equal in the upper bathypelagic and hadal waters. These clusters were affiliated with almost all major prokaryotic and microeukaryotic groups. Plentiful microbial groups with both positive and negative relations were clustered together, suggesting that microbial associations in the trench were diverse and complex. Compared with the hadal waters, more bacterial cluster components with pronounced associations occurred in the upper bathypelagic waters. Overall, our results demonstrated that the complexity of the microbial network differed between the upper bathypelagic and hadal waters in the trench, with different microbial taxa contributing to the modularity of the microbiome network and microbial associations.

The top 10 ASVs ranked according to their relative abundance, degree, and betweenness centrality varied among microbial networks (Table S3). For example, the high-degree nodes (i.e., hub species) in the upper bathypelagic community network were primarily associated with Chloroflexi (*SAR202_clade*), Alphaproteobacteria, and Thermoplasmatota (*Marine_Group_II*) (Table S3). The nodes possessing high betweenness centrality were affiliated to Alphaproteobacteria (*AEGEAN-169_marine_group* and *Roseovarius*), Chloroflexi (*SAR202_clade*), Cyanobacteria (*Prochlorococcus_MIT9313*), Gammaproteobacteria (*Oleibacter* and *Alteromonas*), and Actinobacteriota (*Mycobacterium*) (Table S3). These nodes with high degree (“hub species”) or betweenness centrality (“gatekeepers”) were crucial to the structure and persistence of the microbial networks within the Mariana Trench, as they literally hold the network together. Furthermore, the most abundant microbial taxa differed from the “hub species” (with high degree) and “gatekeepers” (species with high betweenness centrality) in the trench.

### Stability of microbiomes

**Fig 7:**
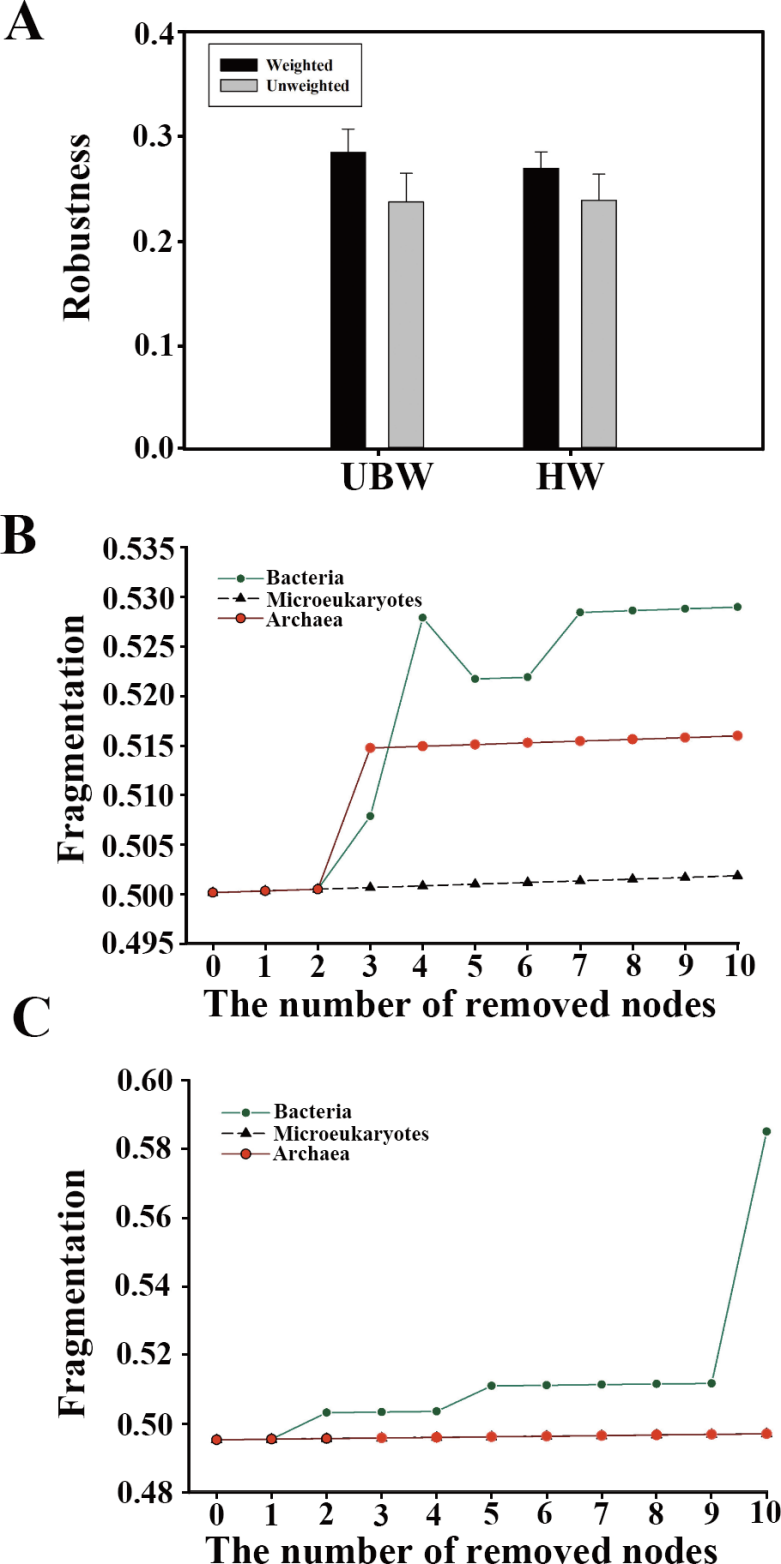
Stability of microbiomes in the trench. Robustness (**A**) measured as the proportion of taxa remained with 50% of the taxa randomly/target removed from each of the network in the UBW and HW. The fragmentations of microbial networks with consecutive removal of 10 nodes with the highest betweenness centrality for bacterial, archaeal, and microeukaryotic communities in the upper bathypelagic (**B**) and hadal (**C**) oceans.

To further explore the stability of microbiomes in the Mariana Trench, the network robustness, fragmentation, and ratio of negative links were calculated and compared (Fig. 7; Table S2). Upon random species loss or targeted removal of module hubs, no significant difference was detected between upper bathypelagic and hadal microbiome robustness in the trench (*P* > 0.01) (Fig. 7A). Similarly, the proportion of negative links in the microbiome networks was also comparable (Table S2), suggesting adaption and coevolution of microbiomes across depths. Consistent stability from the surface to the hadal waters of the trench demonstrates that the vertical gradients in the trench did not destabilize the organization of microbiomes after long-term coevolution and adaption.

After we iteratively removed the top 10 nodes with high betweenness centrality, network fragmentation (*f*) responded differently in upper bathypelagic vs. hadal waters. In upper bathypelagic waters, the fragmentation (*f*) increased sharply after the top three bacterial or archaeal taxa were removed, with no changes in hadal water networks until the top 10 bacterial taxa were lost. Thus, we concluded that (1) compared with microeukaryotes, bacteria and archaea contribute to the upper bathypelagic water microbiome network stability and (2) fragmentation (*f*) of hadal water microbiomes only responded to loss of the top bacterial hub species rather than archaea or microeukaryotes.

## DISCUSSION

### Microbiomes along the depth gradients

Bacterial, archaeal, and microeukaryotic communities displayed consistent alpha diversity and occupied similar niche breadth along the vertical gradients from the surface to the hadal waters (Table 1). Similarly, J. Tian et al. (14) have discovered a uniform bacterial composition from the upper bathypelagic to the hadal waters. Distribution of bacterial communities in the hadal waters was distinct from those in the abyssal waters (4). The constant niche breadth of prokaryotes and microeukaryotes further demonstrates the diverse microbiomes inhabiting the trench from the upper bathypelagic and hadal waters. Our results suggest that the microbiomes are well adapted to the hadal biosphere niche/environments and their diversity can be replenished by species at various depths, ranging from the surface to the hadal waters.

Vertical gradients have a more pronounced effect on bacterial community composition compared with archaea and microeukaryotes, indicating that different microbiomes in the Mariana Trench employ distinct adaptation and survival strategies in response to vertical heterogeneity. For example, protist communities are more sensitive to species sorting and environmental selection than bacterial communities, as protists have a more limited tendency to enter dormancy (31). Furthermore, the phylogenetic distance of bacterial and archaeal microbial communities in upper bathypelagic waters is greater than those in hadal waters, suggesting a higher efficiency of convergent evolution in prokaryotic microbiomes adapting to the constant and extreme hadal environment.

The depth and temperature gradients were found to influence all microbiome distribution with varied strengths. In addition, phosphorus and pH strongly influenced the dynamics of microeukaryotes and archaea, respectively. This pattern is consistent with previous studies (15, 32, 33), demonstrating vertical gradients as the most important environmental factor shaping the distribution of microbial communities in the Mariana Trench. Hadal environments, distinguished by their elevated hydrostatic pressure and isolated from the hydro-topography, are comparable to abyssal oceans in terms of physical and chemical conditions such as temperature, salinity, dissolved oxygen, and nutrients (34). Distinct microbial taxa coexist in the trench from the upper bathypelagic to the hadal oceans, exhibiting strong metabolic plasticity and redundancy, for example, some hadal bacteria in the Mariana Trench have been isolated and proliferated under normal temperatures and pressures (35). These findings support the “size-plasticity” hypothesis: smaller organisms (bacteria) are less environmentally filtered than larger organisms (microeukaryotes), as smaller organisms are more likely to exhibit plasticity in metabolic abilities and possess greater environmental tolerance (36).

### Stochastic process influencing microbiome assembly

The vertical gradients of the Mariana Trench resulted in the decrease of stochasticity of bacterial and archaeal community assembly, while the stochasticity for microeukaryotic communities remained relatively consistent from the upper bathypelagic to the hadal oceans. The drift arising from stochastic differences in population size, proliferation, and death rates (37) may exhibit differential influences on prokaryotic and eukaryotic metacommunity dynamics and assembly (38). Bacteria and archaea generally possess larger population sizes than microeukaryotes and are thus less affected by drifting (39), which could potentially influence the strength of dispersal limitation that operates alongside drift (40). In addition, differences in selective grazing and trophic levels between prey, degrader (bacteria and archaea), and predator (protists) may also contribute to the varied contribution of stochastic processes to multi-domain microbiomes (41).

Vertical connectivity in relation to dispersal limitation may play a less critical role in microeukaryotes due to their weaker dispersal capabilities compared with bacterial communities. For example, microeukaryotes were found to be more governed by species sorting than dispersal limitation compared with bacterial communities in the East China Sea (39). These distinct patterns can be attributed to differences in the influences of the stochastic process on prokaryotic and eukaryotic microbial communities. The relative impact of dispersal limitation on bacterial communities increased with depth, while the relative influence of species sorting is enhanced for microeukaryotes in the trench (39).

### Community complexity and stability of multiple-domain microbiomes

The difference between upper bathypelagic and hadal oceans may cause distinct specialization or environmental selection mechanisms/processes for bacteria, archaea, and microeukaryotes in the Mariana Trench. The co-occurrence networks of cross-domain microbiome associations between upper bathypelagic and hadal waters exhibited more commonalities with consistent complexity, robustness, and the ratio of negative links. However, different major groups and taxa performed various combinations of species and relationships, which might represent specific strategies for adaptation to distinct habitats along vertical gradients. The varied contribution of key species from different domains to community integrity confirmed the inference of niche and functional redundancy, where different taxa possibly share the same ecological functions in the upper bathypelagic and hadal waters.

The consistent complexity and modularity observed in upper bathypelagic and hadal waters also suggest that microbial relations and cross-module associations among taxa are likely to be prevalent in the Mariana Trench. According to MacArthur’s argument (42), the complexity of ecosystems contributes to their stability. Our results demonstrate that the vertical gradients did not destabilize the organization of microbiome communities in the Mariana Trench after long-term coevolution and adaption. Environmental variations have been observed to affect microbial network complexity and stability in soil environments (27), where climate warming enhances soil microbial network complexity and stability. Considering the potential influences of climate change and anthropogenic activities, the accumulation of marine snow, marine organism excrement, and anthropogenic pollutants (i.e., anthropogenic antibiotic pollutants) driven by settling mechanisms due to the extreme vertical gradients will significantly affect the association of microbiomes in the Mariana Trench (24, 25).

The variation of microbial fragmentations can be considered a function of complex evolutionary forces and ecological strategies of microbiomes, reflecting the interplay of environmental, biological, and historical factors in the deepest ocean. The microbiome association may be influenced by multiple ecological and evolutionary mechanisms. Given that the average ocean depth is about 4,000 m and the physicochemical properties of the oceanic water column remain relatively constant at great depths, this implies that there may be abundant prokaryotic and eukaryotic microbes that have co-evolved to survival and adaptative strategies (e.g., to water pressure) for inhabiting rarely explored deep-sea habitats. Compared with archaea and microeukaryotes, bacteria and archaea contribute greatly to the stability of Mariana Trench microbiome networks, especially in upper bathypelagic waters. Similarly, the essential roles of predominant heterotrophs in recycling macromolecules and utilizing various carbon sources (e.g., peptides, hydrocarbons, carbohydrates, and aromatic compounds) are demonstrated in the Mariana Trench (43 – 45). The trench V-shaped topography enables the collection of particulate organic matter and heavy metals, such as selenium and arsenic, from the overlying water column, abyssal seafloor, and Earth’s upper crust (43, 46). These accumulation effects result in a greater content of organic matters and higher heterotrophic microbial degradation activities at the bottom axis than in adjacent slope sites in the trench (47). In addition, the functional redundancy that multiple bacterial taxa encode the same biological function (44, 48) and cross-stress adaptation behaviors for coping with distinct environmental conditions (49) are also reported in the trench and other environments.

This study showed how vertical gradients affect the assembly, organization, and community stability of microbiomes in the deepest trench on Earth. The long-term co-evolution of microbiomes leads to stable associations in which bacterial contributions are substantial. Vertical gradients are a persistent and strong environmental filtering factor, previously reported to act as natural barriers that separate microbial communities and facilitate their differentiation through random genetic drift or adaptation (50). Although our relatively small sample size is due to the challenges of deep-sea sampling, the significant influence of deep stratification can be observed through the analysis of distribution, evolutionary characteristics, and network stability. Further investigations of samples below 10,000 meters, which were limited during this cruise, will be conducted in future studies to provide deeper insights. From a biogeography perspective, our results highlight the importance of considering the vertical structures of hydrographic conditions and the combined contribution of multi-domain microbiomes for studying metacommunity interactions in marine ecosystems. From the perspective of the whole microbial loop in the trench, community association remains relatively constant to maintain the stability and functioning of the entire microbial ecosystem. Our results represent the first comparison of microbiome network stability between the upper bathypelagic and hadal oceans in the deepest habitats on Earth, highlighting the principles of microbiome organization in the trench: although taxa are habitat specific, primary community association properties and stability can be conserved.

## MATERIALS AND METHODS

### Sample collection and characterization

The vertical samples were collected at a site above the Challenger Deep of the Mariana Trench (11.38°N, 142.30°E) on an oceanography survey at south-central western Pacific Ocean in January 2016. Sample collection and measurements of environmental factors followed the protocols described in previous studies (13, 14). Briefly, seawater samples are from water depths of 2, 500, 1,000, 1,759, 3,699, 5,367, 7,200, and 8,727 m. Surface water (~2  m deep) was collected by using a submerged pump. The deeper water samples were collected by using a custom-designed 8–12-L Niskin bottle system equipped with a conductivity-temperature-depth (CTD) device down to 8,727  m below the surface. Depth, temperature, and salinity were continuously recorded by the CTD. For each depth, 2 L of water was prefiltered through a 200-µm pore size mesh and then immediately filtered through a 3 µm (pore size), 47-mm diameter polycarbonate filter (with a gentle vacuum pressure < 25 cm Hg), followed by a 0.22-µm filter (Whatman, Piscataway, NJ, USA). Each filter was carefully placed into a 5-mL tube containing 2 mL of lysis buffer (50 mM Tris-HCl, 1.0 mM EDTA, 150 mM NaCl, and 0.1% SDS). The samples were quickly frozen in liquid nitrogen and stored at −80°C until DNA extraction. Depth, temperature, DO were measured on board by the sensors mounted on the CTD equipment while nutrient concentrations (PO_4_^3−^, NO_3_^−^, NO_2_^−^, NH_4_^+^, and SiO_3_^2−^) were subsequently analyzed with a continuous-flow auto-analyzer (AA3, Seal Analytical Inc., Southampton, UK).

### DNA extraction, sequencing, and analysis

DNA extraction was performed according to a previous protocol (51) by using phenol- chloroform-isoamyl (1:1) extraction combined with precipitation and washing procedures (52). The DNA quantity of the samples was assessed using a NanoDrop 2000 spectrophotometer (Thermo Fisher Scientific, Waltham, Massachusetts, USA). Extracted DNA samples were qualified for high-throughput sequencing of bacteria, archaea, and microeukaryotes. Specifically, the V4-V5 region of the archaeal 16S rRNA gene, the V3-V4 region of the bacterial 16S rRNA gene, and the V4 region of 18S rRNA genes were amplified using primers 524F (5′-TGYCAGCCGCCGCGGTAA-3′)/958R (5′-YCCGGCGTTGAVTCCAATT-3′), 338F (5′-ACTCCTACGGGAGGCAGCA-3′)/806R (5′-GGACTACHVGGGTWTCTAAT-3′)(53), and 3NDF (5′-GGCAAGTCTGGTGCCAG-3′)/V4_euk_R2 (5′-ACGGTATCT(AG)ATC(AG)TCTTCG-3′)(54), respectively. High-throughput sequencing was conducted on the Illumina MiSeq platform following the PE300 protocol (MajorBio, China).

The raw sequence reads were processed and analyzed with QIIME (Quantitative Insights into Microbial Ecology) 2_2021.4 (55), with the following steps: forward and reverse sequence files were spliced, low-quality reads were removed (quality score < 25), and primers and extreme data were deleted. DNA concentration and quality, sequencing depth, quality of raw reads, and further results all demonstrated that the samples were qualified for downstream analysis. ASVs were determined using the DADA2 algorithm (56). ASVs detected only in one sample or with less than five copies across all samples were removed. A representative sequence of each ASV was selected for taxonomic identification according to the Silva database (v138) (57). After quality control and filtering, high-quality sequences were clustered into 1,219 ASVs for bacteria, 638 ASVs for archaea, and 1,039 ASVs for microeukaryotes, of which all were rarified to 16,000 sequences across samples. The rarefaction curves approached saturation for the seawater and sediment samples (Fig. S1), which indicated that the sequencing had sampled enough microbial diversity across samples. Sequences of archaeal and bacterial 16S rRNA genes generated in this study were deposited in the Sequence Read Archive under the accession number SRP150142, and the 18S rRNA raw reads were deposited under the accession number SRP148847.

### MRT analysis

To determine how the Mariana Trench microbiomes respond to environmental gradients, MRT was used to evaluate the hierarchical effects of the vertical gradients on the microbiomes (58). It was determined and generated by the R package “mvpart” (R version 3.6.1), and the divisions in the MRT were determined by cross-validation.

### Statistical analyses on sequence data

The statistical analyses and measures applied in this study are briefly described in Table S1, including the neutral community model, phylogenetic distance, SES.MNTD, βNTI, niche breadth, beta diversity, co-occurrence networks, fragmentation, and robustness. The details of conducting these statistical analyses are available in the supplemental materials. Differences between the alpha diversity of total, abundant, and rare subgroups of bacteria, archaea, and microeukaryotes were compared using a one-way and two-way ANOVA (*P* = 0.01) with depths and sampling pore sizes (free-living and particle-associated communities). Tukey’s *post hoc* tests were used to test the hypothesized differences. Differences were ensured to be qualified and then determined if statistically significant (*P* ≤ 0.01). In order to describe variations of microbiomes over depths and pore sizes, NMDS was performed based on four distance matrices: Jaccard, Bray-Curtis, Unweighted-UniFrac, and Weighted-UniFrac. The NMDS analyses were performed using “metaMDS” functions in the vegan R package (R version 3.6.0). Relationships between diversities of microbial communities and depth were tested using Spearman’s rank correlation coefficient with the corrplot R package (R version 3.6.0).

## SUPPLEMENTAL MATERIAL

Additional experimental details spectrum.02110-23-s0001.docx Materials and methods; Figures S1 and S2.

Table S1 spectrum.02110-23-s0002.docx Statistical analyses and measurements applied in this study.

Table S2 spectrum.02110-23-s0003.docx Table S2. microbial co-occurrence networks.

Table S3 spectrum.02110-23-s0004.txt Top 10 nodes based on abundance/degree/betweenness centrality in the shallow and deep co-occurrence networks.

Table S4 spectrum.02110-23-s0005.docx Metadata on environmental factors from surface to the hadal zone in the Mariana Trench.

ASM does not own the copyrights to Supplemental Material that may be linked to, or accessed through, an article. The authors have granted ASM a non-exclusive, world-wide license to publish the Supplemental Material files. Please contact the corresponding author directly for reuse.
