# Supplementary material for: Depth shapes microbiome assembly and network stability in the Mariana Trench
Source: Microbiol Spectr. 2023 Dec 12;12(1):e02110-23. doi: 10.1128/spectrum.02110-23 (PMC10783068; doi:10.1128/spectrum.02110-23)
Supplement: Additional experimental details — Materials and methods; Figures S1 and S2. [file spectrum.02110-23-s0001.docx]

# Depth shapes microbiome assembly and network stability in the Mariana Trench

Yi Li^1,2^, Jinjun Kan^3*^, Feilong Liu^1,2^, Kaiyue Lian^1,2^, Yantao Liang^1,2^, Hongbing Shao^1,2^, Andrew McMinn^4^, Hualong Wang^1,2*^, Min Wang^1,2*^

^1^College of Marine Life Sciences, Institute of Evolution and Marine Biodiversity, Frontiers Science Center for Deep Ocean Multispheres and Earth System, and Key Lab of Polar Oceanography and Global Ocean Change, Ocean University of China, Qingdao, China

^2^UMT-OUC Joint Center for Marine Studies, Qingdao, China

^3^Microbiology Division, Stroud Water Research Center, Avondale, PA, USA

^4^Institute for Marine and Antarctic Studies, University of Tasmania, Hobart, Tasmania, 7005, Australia

**Correspondence:** Min Wang ([mingwang@ouc.edu.cn](mailto:mingwang@ouc.edu.cn)) ; Jinjun Kan ([jkan@stroudcenter.org](mailto:jkan@stroudcenter.org)); Hualong Wang (wanghualong@ouc.edu.cn)

## Materials and methods

### Neutral community model

To determine the potential importance of stochastic processes on the assembly of Mariana Trench microbiomes, neutral community model (NCM) has been applied to calculate the relationships between the ASV detection frequency and their relative abundance (33). The model used here was an adaptation of the neutral theory adjusted to be suitable for the large microbial populations (34-37). In general, the model predicts that in a meta-community, abundant taxa are widespread since they are more likely to disperse by chance among different sampling sites, whereas rare taxa are more likely to be lost due to ecological drift (i.e., the stochastic loss and replacement of individuals). In this model, the parameter Nm determines the correlation between occurrence frequency and regional relative abundance, with N describing the metacommunity size and m being the immigration rate. Nm is an estimate of dispersal between communities, and the R^2^ represents the overall fit to the neutral model (33). Calculation of 95% confidence intervals around all fitting statistics was done by boot-strapping with 1,000 bootstrap replicates. The ASVs from each dataset were subsequently separated into three partitions depending on whether they occurred more frequently than (above partition), less frequently than (below partition), or within (neutral partition) the 95% confidence interval of the NCM predictions. To analyze deviations from the NCM predictions, we compared the composition, diversity, and calculated estimated migration rate (m) of neutral and non-neutral (above and below) partitions of microeukaryotes, bacteria and archaea. All computations were performed in R package “Hmisc”, “minpack.lm” and “stats4”(version 4.1.2) (38).

### Phylogenetic distance, niche breadth, and beta diversity

Mean nearest taxon distance (MNTD) is the mean distance between each species within a community and its closest relative. MNTD reflects phylogenetic structure closer to the tips and requires the phylogeny to be represented as a phylogenetic distance matrix (39). To estimate the phylogenetic clustering of shallow and deep layer bacterial, microeukaryotic and archaeal communities, we calculated a standardized index using the mean nearest taxon distance (SES.MNTD) by means of the “ses.mntd” function in the “picante” package of R (39). Positive values of SES.MNTD indicate phylogenetic evenness, i.e., species within the community are more distantly related than expected by chance. Negative values of SES.MNTD indicate phylogenetic clustering, i.e., species within the community are more closely related than expected by chance.

Null model-based Bray-Curtis-based β-nearest taxon index (βNTI) was applied to calculate the differences in taxonomic and phylogenetic diversity using the “picante” package of R. If βNTI < −2 or βNTI >2, deterministic processes govern the community assembly, with distinctly more (i.e., variable selection; βNTI > 2) and less (i.e., homogeneous selection; βNTI < –2) phylogenetic turnover than expected. Whereas │βNTI│ < 2 denote that stochastic rather than deterministic is dominating the microbiome assembly (40, 44).

Niche breadth refers to the sum of the diverse resources that a population (or other biological unit) uses in a community. In the case of less available resources, niche breadth is generally increased to allow adequate resources to be available to the population. In an environment with abundant available resources, selective utilization of resources can result in narrow niche width (45). To reveal the taxa sorting and dispersal patterns, we estimated Levins' niche breadth index based on Shannon-Wiener index (46) for microbial communities according to the formula (47). The analysis was conducted using the R package “spa” (48).

Beta diversity, also known as between-habitat diversity, refers to species composition between samples (49, 50). Common beta diversity metrics include Bray–Curtis distance (abundance without phylogeny), Jaccard distance (presence and absence of ASVs without phylogeny), unweighted UniFrac distance (presence and absence of ASVs with phylogeny), and weighted UniFrac distance (abundance of ASVs with phylogeny). Beta-diversity measures can be generated with QIIME2.

### Co-occurrence network analysis

Co-occurrence networks of multi-domain microbiomes including bacteria, archaea and microeukaryotes were constructed at the ASV level. All network constructions were performed in R (version 4.1.0) and corresponding codes were adapted from GitHub (https://github.com/ryanjw/co-occurrence) (36).

Network visualization and topological analysis were carried out in Gephi (version 0.9.2). Nodes and edges in microbial co-occurrence networks represent microbial taxa and statistically significant associations between nodes, respectively. The topological properties of microbial networks were calculated with indexes including components (those separated subgraphs in a network), degree (number of connections), average clustering coefficient (the degree that nodes tend to cluster together), modularity, network diameter (longest distance), clusters (a group of nodes with higher number of within-cluster edges than between-cluster edges), graph density, average path length, proportions of positive and negative correlations, and network fragmentation (*f*) (51-54). The nodes with high degree or high betweenness centrality (how well a node is interacting simultaneously with different compartments of the network, potentially “gatekeepers”) are crucial for ecological network structure and persistence because they literally hold the network together (55-58). Microbial networks were also analyzed at species level to mine more universal patterns.

Network complexity (including network size, connectivity, average clustering coefficient, relative modularity) and stability (e.g., robustness, fragmentation, proportion of negative correlations, etc.) of microbiomes were primarily characterized based on network properties and further statistical analysis. The *f* was calculated as the ratio of the number of disconnected subgraphs (CL) to the overall number of nodes (N) in each network as log(CL)/log(N) (59). The *f* ranges from 0 to 1, and closer to 1 represents more fragmented and less stable networks. Loss of “gatekeepers” (i.e., nodes with high betweenness centrality) contributes disproportionately to network fragmentation, suggesting high fragility of these networks upon selective removal of species (59, 60). Therefore, the network stability was further tested by recording *f* upon iteratively removing the top 10 “gatekeeper” nodes with the most abundance, betweenness centrality, and degree (59, 61).

Robustness of network is defined as the proportion of the remaining species in this network after random or targeted node removal (62, 63). For simulations of random species removal, a certain proportion of nodes was randomly removed. For simulations of targeted removal, certain numbers of module hubs were removed. To test the effects of species removal on the remaining species, we calculated the abundance-weighted mean interaction strength (wMIS) of node *i* as

wMIS*i* = $\frac{\sum_{j\neq i} bjsij}{\sum_{j\neq i} bj}$

where *b_j_* is the relative abundance of species *j* and *s_ij_* is the association strength between species *i* and *j*, which is measured by Pearson correlation coefficient. Thus, in this study, s*_ij_* = s*_ji_*. After removing the selected nodes from the network, if wMIS*_i_* = 0 (all the links to species i have been removed) or wMIS*_i_* < 0 (not enough mutualistic association between species i and other species for its survival), node *i* was considered extinct/isolated and thus removed from the network. This process continued until all species had positive wMISs. The proportion of the remaining nodes was reported as the network robustness. We measured and reported the robustness when 50% of random nodes or five module hubs were removed.


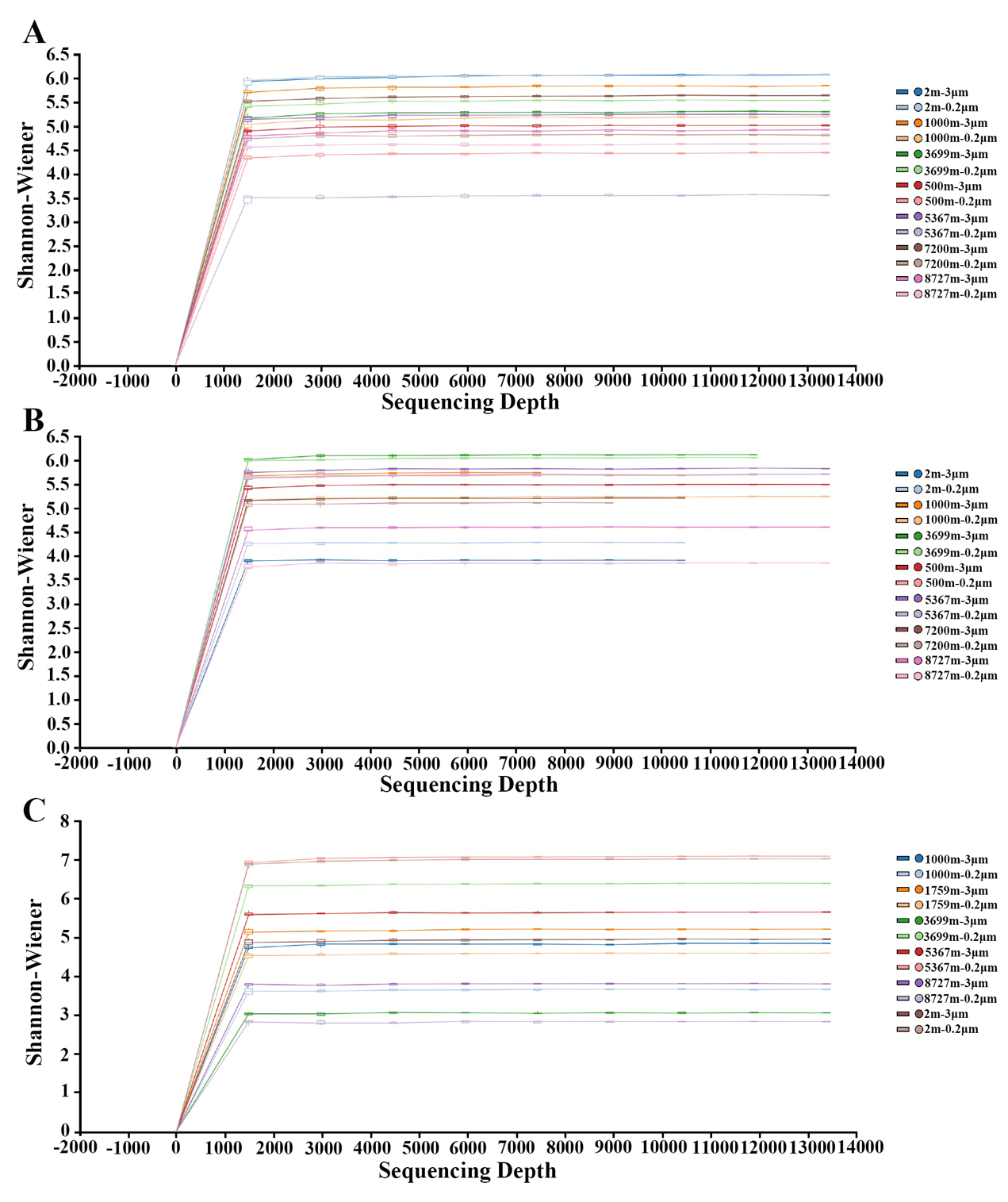


**Figure S1.** Rarefaction curves of observed ASVs. A, bacterial samples; B, archaeal samples. C, microeukaryotic samples.


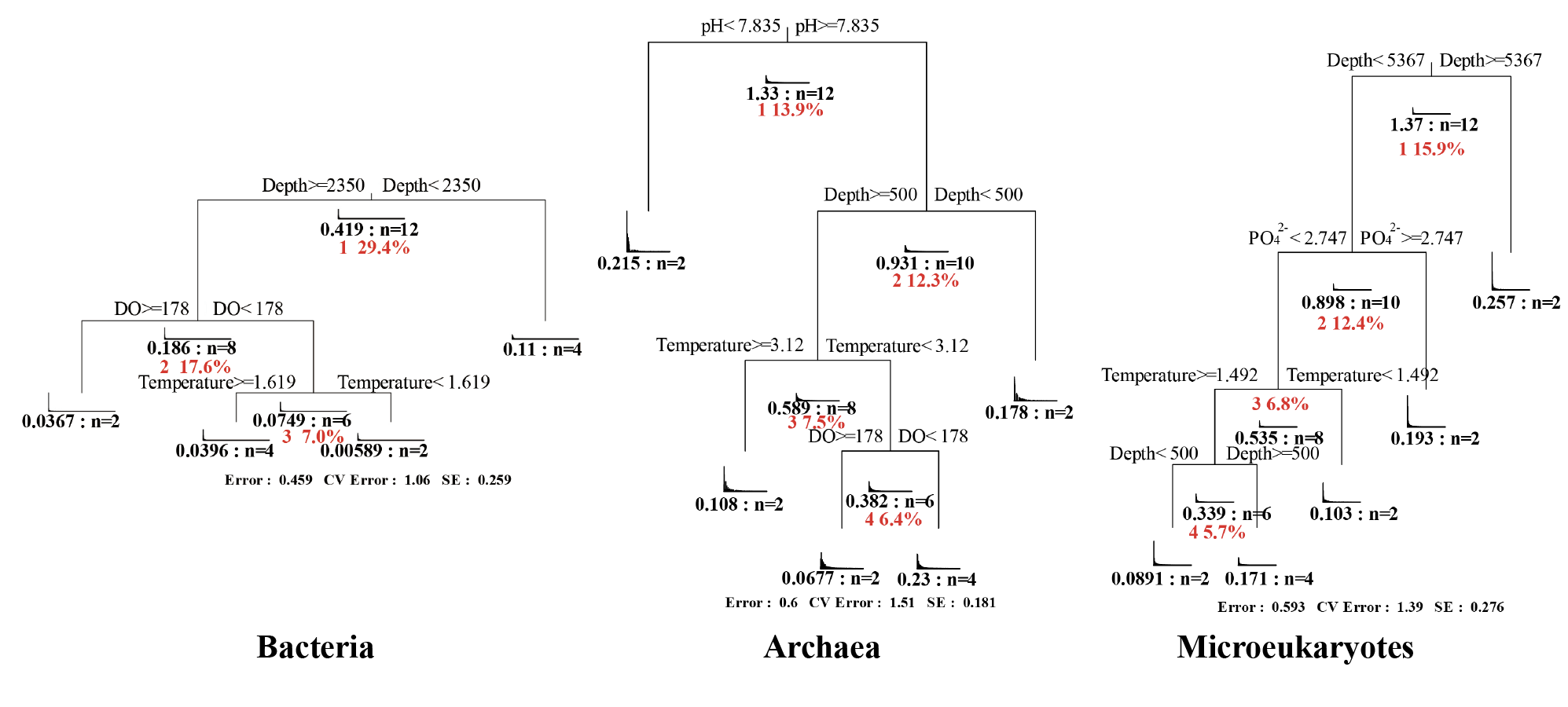


**Figure S2.** Evaluating the hierarchical effect of environmental variations on the microbiomes by Multivariate regression tree.

**Table S1.** Statistical analyses and measurements applied in this study

**Table S2.** Topological properties of microbial co-occurrence networks

**Table S3.** Top 10 nodes based on abundance/degree/betweenness centrality in the shallow and deep co-occurrence networks

**Table S4.** Metadata on environmental factors from surface to the hadal zone in the Mariana Trench
