# Supplementary material for: Depth shapes microbiome assembly and network stability in the Mariana Trench
Source: Microbiol Spectr. 2023 Dec 12;12(1):e02110-23. doi: 10.1128/spectrum.02110-23 (PMC10783068; doi:10.1128/spectrum.02110-23)
Supplement: Table S1 — Statistical analyses and measurements applied in this study. [file spectrum.02110-23-s0002.docx]

| Table S1. Statistical analyses and measurements applied in this study. | |
| --- | --- |
| Statistical analysis | Meanings |
| Neutral community model | Determine the potential importance of stochastic processes on the assembly of Mariana Trench microbiomes. |
| Phylogenetic distance | Characterize the phylogenetic community composition within each sample. |
| SES.MNTD | Describe the mean phylogenetic distance among individuals. |
| βNTI | Null model-based Bray-Curtis-based β-nearest taxon index (βNTI) was applied to calculate the differences in taxonomic and phylogenetic diversity. |
| Niche breadth | Reveal the taxa sorting and dispersal patterns. |
| Beta diversity | Reveal the species composition between samples. |
| Co-occurrence networks | Visualize the microbial correlations. |
| Fragmentation | Calculated as the ratio of the number of disconnected subgraphs (CL) to the overall number of nodes (N) in each network as log(CL)/log(N). The fragmentation ranges from 0 to 1, and closer to 1 represents more fragmented and less stable networks. |
| Robustness | defined as the proportion of the remaining species in this network after random or targeted node removal. |
|  |  |
