# Supplementary material for: Depth shapes microbiome assembly and network stability in the Mariana Trench
Source: Microbiol Spectr. 2023 Dec 12;12(1):e02110-23. doi: 10.1128/spectrum.02110-23 (PMC10783068; doi:10.1128/spectrum.02110-23)
Supplement: Table S2 — microbial co-occurrence networks. [file spectrum.02110-23-s0003.docx]

| Table S2. Topological properties of microbial co-occurrence networks. | | | | | | | | | | | | |
| --- | --- | --- | --- | --- | --- | --- | --- | --- | --- | --- | --- | --- |
|  | Groups | Nodes | Edges | Average degree | Network diameter | Graph density | Modularity | Components | Average Clustering Coefficient | Average path length | Number of Clusters | Percentage of negative correlations (%) |
| Upper bathypelagic waters | All | 483 | 7575 | 31.4 | 15 | 0.065 | 0.61 | 22 | 0.85 | 3.193 | 25 | 1.81 |
|  | Delete the ten highest betweenness nodes of bacteria | 473 | 6948 | 29.4 | 8 | 0.062 | 0.638 | 26 | 0.864 | 1.751 | 27 |  |
|  | Delete the ten highest betweenness nodes of microeukaryotes | 473 | 5814 | 24.6 | 15 | 0.052 | 0.704 | 22 | 0.859 | 3.388 | 25 |  |
|  | Delete the ten highest betweenness nodes of archaea | 473 | 7084 | 30 | 15 | 0.063 | 0.619 | 24 | 0.863 | 3.135 | 27 |  |
| Hadal waters | All | 467 | 6808 | 29.2 | 12 | 0.063 | 0.729 | 21 | 0.852 | 2.822 | 25 | 1.66 |
|  | Delete the ten highest betweenness nodes of bacteria | 457 | 6147 | 26.9 | 10 | 0.059 | 0.744 | 36 | 0.856 | 1.698 | 37 |  |
|  | Delete the ten highest betweenness nodes of microeukaryotes | 457 | 5833 | 25.5 | 12 | 0.056 | 0.75 | 21 | 0.857 | 2.889 | 25 |  |
|  | Delete the ten highest betweenness nodes of archaea | 457 | 6368 | 27.9 | 12 | 0.061 | 0.711 | 21 | 0.85 | 2.858 | 25 |  |
