# Supplementary material for: Depth shapes microbiome assembly and network stability in the Mariana Trench
Source: Microbiol Spectr. 2023 Dec 12;12(1):e02110-23. doi: 10.1128/spectrum.02110-23 (PMC10783068; doi:10.1128/spectrum.02110-23)
Supplement: Table S4 — Metadata on environmental factors from surface to the hadal zone in the Mariana Trench. [file spectrum.02110-23-s0005.docx]

| Table S4. Metadata on environmental factors from surface to the hadal zone in the Mariana Trench. | | | | | | | | | | | |
| --- | --- | --- | --- | --- | --- | --- | --- | --- | --- | --- | --- |
| Depths | Pore size | DO | pH | Phosphorus | Nitrate | Nitrite | Ammonium | Silicate | | Temperature | Salinity |
| 2m | 0.2μm | 205.22 | 8.30 | 0.12 | 0.40 | 0.06 | 1.81 | 1.36 | | 28.12 | 34.36 |
| 2m | 3μm | 205.22 | 8.30 | 0.12 | 0.40 | 0.06 | 1.81 | 1.36 | | 28.12 | 34.36 |
| 1000m | 0.2μm | 86.48 | 7.85 | 2.84 | 41.47 | 0.05 | 0.72 | 109.22 | | 4.48 | 34.54 |
| 1000m | 3μm | 86.48 | 7.85 | 2.84 | 41.47 | 0.05 | 0.72 | 109.22 | | 4.48 | 34.54 |
| 1759m | 0.2μm | 121.95 | 7.89 | 2.65 | 38.10 | 0.04 | 1.19 | 139.81 | | 2.39 | 34.61 |
| 1759m | 3μm | 121.95 | 7.89 | 2.65 | 38.10 | 0.04 | 1.19 | 139.81 | | 2.39 | 34.61 |
| 3699m | 0.2μm | 159.19 | 7.93 | 2.54 | 27.73 | 0.03 | 1.33 | 161.65 | | 1.48 | 34.68 |
| 3699m | 3μm | 159.19 | 7.93 | 2.54 | 27.73 | 0.03 | 1.33 | 161.65 | | 1.48 | 34.68 |
| 5367m | 0.2μm | 180.94 | 7.92 | 2.42 | 43.10 | 0.02 | 0.48 | 150.00 | | 1.50 | 34.69 |
| 5367m | 3μm | 180.94 | 7.92 | 2.42 | 43.10 | 0.02 | 0.48 | 150.00 | | 1.50 | 34.69 |
| 8727m | 0.2μm | 173.24 | 7.82 | 2.43 | 35.30 | 0.06 | 0.61 | 142.72 | | 2.01 | 34.69 |
| 8727m | 3μm | 173.24 | 7.82 | 2.43 | 35.30 | 0.06 | 0.61 | 142.72 | | 2.01 | 34.69 |
|  |  |  |  |  |  |  |  | |  |  |  |
